# Compritol-Based Nanostrucutured Lipid Carriers (NLCs) for Augmentation of Zolmitriptan Bioavailability via the Transdermal Route: In Vitro Optimization, Ex Vivo Permeation, In Vivo Pharmacokinetic Study

**DOI:** 10.3390/pharmaceutics14071484

**Published:** 2022-07-18

**Authors:** Doaa H. Hassan, Joseph N. Shohdy, Doaa Ahmed El-Setouhy, Mohamed El-Nabarawi, Marianne J. Naguib

**Affiliations:** 1 Department of Pharmaceutics, College of Pharmaceutical Sciences and Drug Manufacturing, Misr University for Science and Technology (MUST), Oct. 6, Giza 12566, Egypt; doaa.hassan@must.edu.eg; 2 Department of Industrial Pharmacy, College of Pharmaceutical Sciences and Drug Manufacturing, Misr University for Science and Technology (MUST), Oct. 6, Giza 12566, Egypt; joseph.naeem@must.edu.eg; 3 Department of Pharmaceutics and Industrial Pharmacy, Faculty of Pharmacy, Cairo University, Cairo 11562, Egypt; doaa.elsetouhy@pharma.cu.edu.eg (D.A.E.-S.); mohamed.elnabarawi@pharma.cu.edu.eg (M.E.-N.)

**Keywords:** zolmitriptan, transdermal, nanostructured lipid carriers, pharmacokinetic, ex vivo cytotoxicity

## Abstract

Migraine is a severe neurovascular disease manifested mainly as unilateral throbbing headaches. Triptans are agonists for serotonin receptors. Zolmitriptan (ZMP) is a biopharmaceutics classification system (BCS) class III medication with an absolute oral bioavailability of less than 40%. As a result, our research intended to increase ZMP bioavailability by developing transdermal nanostructured lipid carriers (NLCs). NLCs were prepared utilizing a combination of hot melt emulsification and high-speed stirring in a 3^2^ full factorial design. The studied variables were liquid lipid type (X_1_) and surfactant type (X_2_). The developed NLCs were evaluated in terms of particle size (Y_1_, nm), polydispersity index (Y_2_, PDI), zeta potential (Y_3_, mV), entrapment efficacy (Y_4_, %) and amount released after 6 h (Q6h, Y_5_, %). At 1% Mygliol as liquid lipid component and 1% Span 20 as surfactant, the optimized formula (NLC9) showed a minimum particle size (138 ± 7.07 nm), minimum polydispersity index (0.39 ± 0.001), acceptable zeta potential (−22.1 ± 0.80), maximum entrapment efficiency (73 ± 0.10%) and maximum amount released after 6 h (83.22 ± 0.10%). The optimized formula was then incorporated into gel preparation (HPMC) to improve the system stability and ease of application. Then, the pharmacokinetic study was conducted on rabbits in a cross-over design. The calculated parameters showed a higher area under the curve (AUC_0–24,_ AUC_0–∞_ (ng·h/mL)) of the developed ZMP-NLCs loaded gel, with a 1.76-fold increase in bioavailability in comparison to the orally administered marketed product (Zomig^®^). A histopathological examination revealed the safety of the developed nanoparticles. The declared results highlight the potential of utilizing the proposed NLCs for the transdermal delivery of ZMP to improve the drug bioavailability.

## 1. Introduction

Migraine is a severe neurovascular disease manifested mainly as unilateral throbbing headaches accompanied by a variety of neurological symptoms, such as hypersensitivity to sound, light and smell. Among other symptoms, nausea and autonomic, cognitive, emotional and motor disturbances have also been reported [1]. The attack is usually accompanied with intense unilateral pain, in addition to phonosensitivity and photosensitivity [2]. Aura is a group of localized neurologic symptoms (typically visual) that can accompany headaches in up to one-third of patients [3]. The mechanism for migraine development proposes that primary sensory nerve terminals that innervate meningeal blood vessels release vasoactive peptides early in the attack [4]. These peptides produce a dilation of arteries in the meninges, perivascular inflammation and plasma extravasation by activating perivascular trigeminal neurons [5]. These effects are possibly the reasons for the pain felt. Triptans are serotonin receptor agonists that are used to treat migraines [6]. For patients with moderate to severe migraine, they are considered first-line therapy [7]. The introduction of triptans for migraine management was a revolution, despite drawbacks such as short half-life durations for medications such as rizatriptan (2.4 h) [8], eletriptan (5 h) [9], naratriptan (5.5 h) [10], sumatriptan (2 h) [11] and zolmitriptan (2.5 h) [12].

Zolmitriptan (ZMP) is a partial agonist for the 5-HT_1B/1D_ receptor ratified for the treatment of acute migraine attacks [13]. ZMP is classified as a class III drug according to BCS [14] that is characterized by a low permeability and high solubility [15]. It has a pKa value of 9.52, with an elimination half-life of 2.5–3 h [16]. The drug suffers from a low oral bioavailability (≈40%) [16] due to hepatic first pass metabolism. Owing to its low bioavailability and short half-life, the recurrence of migraine attacks is common, which leads to frequent dosing with an increase in the associated side effects [17], namely, nausea, dizziness and even chest symptoms [18]. All of this prompted a search for alternative delivery routes to help boost ZMP bioavailability. Sub-lingual tablets [19], orodispersible tablets [20] and mucoadhesive films [21] are some of the trials for the oral dosing of ZMP. However, the usage of these dosage forms is restricted due to the vomiting and stomach symptoms that migraine patients experience [22]. Other attempts that were reported to enhance ZMP bioavailability were carried out by its encapsulation in solid lipid nanoparticles (SLNs) [23]. On the other hand, Awadeen et al. [14] prepared ZMP-loaded nanostructured lipid carriers (NLCs). Their results revealed the higher efficacy of the formulated nanocarriers in a pharmacodynamic study. Transfersomes [24] and nasal sprays [25] have also been reported to enhance ZMP bioavailability and efficacy, respectively.

Nanostructured lipid carriers (NLCs) are the second generation of lipid nanoparticles, and were created to overcome the drawbacks of solid lipid nanoparticles (SLNs), such as the low drug loading capacity, gelation and reduced stability [26]. Unlike SLNs, which are made entirely of solid lipids (at ambient temperature), NLCs are made up of both liquid and solid lipids. Due to the fact that liquid lipids lower the melting point of the lipid mixture, the solid lipid matrix became less ordered than SLNs, and more flexible [27]. Therefore, a higher degree of imperfections is found, and, consequently, a higher drug loading capacity, especially for lypophilic drugs, is obtained [28]. Like many other lipid-based nanoformulations, NLCs are considered as both biocompatible and biodegradable [29].

The transdermal drug delivery route has significant advantages over the conventional oral route. It can provide more patient compliance [30], especially in patients with vomiting and gastric problems—a common manifestation of migraine—more stable serum drug levels [31], pain-free drug administration, avoiding hepatic first-pass metabolism [32] and drug degradation in the gastrointestinal tract [33], food–drug interaction and reducing side effects [34,35]. Several attempts have been reported for employing NLCs as a nanoparticulate system for transdermal drug delivery [36,37]. Anantaworasakul et al. [37] have formulated NLCs containing capsaicin. The in vitro skin permeation study revealed the higher penetration powers of the prepared NLCs in comparison to both chili extract solution and SLNs. Tetrahydrocurcumin (THC) was also transdermally delivered to treat breast cancer utilizing chitosan-coated NLCs [38]. The prepared lipid carriers significantly enhanced THC cytotoxicity against breast cancer cell lines.

No trials have been recorded, to the best of our knowledge, for the preparation of nano-structured lipid carriers (NLCs) containing zolmitriptan (ZMP) intended for transdermal drug delivery. Thus, this work is directed at recuperating ZMP bioavailability via the preparation and optimization of ZMP-loaded NLCs intended for topical application to deliver the drug systemically. Nine formulae were prepared adopting a 3^2^ full factorial design utilizing Design Expert^®^ software (Ver. 7; Stat-Ease Inc., Minneapolis, MN, USA). Optimization was conducted in terms of the significant constrains, namely, minimizing particles size and PDI values and maximizing zeta potential, entrapment efficiency and amount released after 6 h. The candidate formula was then incorporated into two different gel bases. The prepared gels were then subjected to gel characterization and ex vivo permeation. The optimum gel formula was eventually subjected to a pharmacokinetic study in rabbits in a cross-over design.

## 2. Materials and Methods

### 2.1. Materials

Zolmitriptan (ZMP) was supplied as a gift from Pharmaceutical Industries, EIPICO (Cairo, Egypt). Span 20 (S20), Span 60 (S60), hydroxypropyl methyl cellulose E15 (HPMC) and cellulose membrane (cut off 12,000–14,000 Mw) were procured from Sigma Aldrich Chemical Co. (St. Louis, MO, USA). El-Nasr Pharmaceutical Chemicals Company (Cairo, Egypt) provided Tween 80 (T80) and triethanolamine (TEA). Pemulen^®^ TR-2 was a kind gift from Luna Pharmaceuticals (Cairo, Egypt). Glyceryl behenate (Compritol 888 ATO^®^), linoleoyl polyoxyl-6 glycerides (Labrafil^®^ 2125), caprylocaproyl macrogol-8 glycerides (Labrasol^®^) and glyceryl tricaprylate/caprate (Miglyol^®^ 812) were obtained as a gift from Gattefosse (St-Priest, France). Methanol was provided by Merck (Darmstadt, Germany).

### 2.2. Methodology

#### 2.2.1. Experimental Design

A tailored 3^2^ full factorial design was employed. The design was used to investigate the effect of independent variables on the measured responses of the prepared nano-lipid carriers (NLCs). Design Expert^®^ software (ver. 7; Stat-Ease Inc., Minneapolis, MN, USA) was employed to create the runs with a two-factor, three-level statistical analysis. The studied variables were liquid lipid type (X_1_) and surfactant type (X_2_). The considered formulation variables, together with the anticipated constraints, are shown in Table 1. All of the prepared NLCs contained Compritol^®^ as a solid lipid entity in a concentration of 1%. Three different types of liquid lipids (X_1_) were utilized, namely, Labrafil^®^, Labrasol^®^ and Miglyol^®^, at a concentration of 1%. Both solid lipid and liquid lipids were utilized as builders of the walls of the prepared nanoparticles. Moreover, three different types of surfactants (X_2_) were used as stabilizer, namely, Span 20, Span 60 and Tween 80. The prepared NLCs were evaluated in terms of particle size (PS, Y_1_, nm), polydispersity index (PDI, Y_2_), zeta potential (ZP, Y_3_, mV), entrapment efficacy (EE, Y_4_, %) and amount released after 6 h (Q6h, Y_5_, %). The composition of the nine prepared NLCs is presented in Table 2. The statistical analysis of the observed responses was performed employing analysis of variance (ANOVA) by either main effects or two-factor interaction models at a *p*-value < 0.05. The models were then ratified through determining their R^2^ (adjusted and predicted), adequate precision and lack of fit. A discrepancy between the adjusted and predicted R^2^ values of less than 0.2, an adequate precision of more than 4 and an insignificant lack of fit could suggest the model’s ability to forecast values and pilot the design space. 

#### 2.2.2. Preparation of ZMP Loaded NLCs

NLCs were fabricated utilizing a combination of hot melt emulsification [39], high-speed stirring and ultrasonication method in a 3^2^ full factorial design (Table 2). Briefly, solid lipid and liquid lipid were mixed and heated under moderate stirring at 85 ℃ to form a uniform transparent oil phase [40]. The total amount of solid lipid and liquid lipid was 2% (*w*/*v*). Double distilled water (10 mL) containing SAA (1% *w*/*v*) as a stabilizer and the drug ZMP (12.5 mg) was used as the aqueous phase. Then, the aqueous and the lipid phases were heated separately for 10 min at the same temperature. Finally, the aqueous phase was poured dropwise to the lipid phase and mixed for 10 min at 20,000 rpm with a high-speed magnetic stirrer. The obtained pre-emulsion was then treated by probe sonicator for 15 min (3 min/3 s, on/off and 50% voltage efficiency) (VCX600, Sonics and Materials, Newtown, CT, USA) surrounded by ice bath to prevent excessive heating of the system. The final formulae′s total volume was adjusted to 10 mL. The obtained dispersions were stored at 4 °C for further investigations.

#### 2.2.3. Characterization of ZMP Loaded NLCs

##### Determination of Particle Size (PS, nm), Polydispersity Index (PDI) and Zeta Potential (ZP, mV)

The mean PS and PDI of the prepared NLCs were measured by a Malvern Zetasizer (Zetasizer Nano ZS, Malvern Instruments, Worcestershire, UK) utilizing dynamic light scattering technique. The measurements were performed after dilution (10 folds) by double distilled water [32,41]. The electrophoretic movement of the particles in the electrical field was monitored during the ZP evaluation. The electrophoretic mobility was converted to ZP (mV) using Smoluchowski equation. All measurements were performed in triplicate ± SD.

##### Determination of Entrapment Efficiency (EE, %)

The EE of ZMP-loaded NLCs was assessed using the direct technique, which involved spectrophotometrically (UV-1601 PC Shimadzu spectrophotometer, Kyoto, Japan) quantifying the amount of entrapped drug (present in the sediment) at λ_max_ 283 nm [42]. The prepared NLCs were separated from the supernatant by centrifugation utilizing a cooling centrifuge (Sigma 3–30 KS, Roedermark, Germany) at 20,000 rpm for 1 h and 4 °C. All of the determinations were carried out in triplicate. Results are expressed as mean values ± SD. The following equation was employed:

$$EE\%=\left(\frac{ED}{TD}\right)\times 100$$

where *ED* is the concentration of entrapped drug and *TD* is the total drug concentration. 

##### Determination of Amount of Drug Released after 6 h (Q6h)

The dissolution profiles of the prepared NLCs and the amount of the drug released after 6 h (Q6h) were determined utilizing USP dissolution apparatus I for 6 h at 35 °C ± 0.5 by applying the membrane diffusion technique [32]. Before use, the cellulose membrane was soaked in double distilled water overnight [43]. The soaked cellulose membrane was then placed at the end of cylindrical plastic tubes with a permeation area of 3.14 cm^2^. The opposite ends of the tubes were linked to the shaft of the USP dissolution apparatus instead of the baskets. Samples of 2 mL of the prepared NLCs formulae (containing 2.5 mg of ZMP) were loaded in the donor compartment [44]. The baskets were then immersed in 50 mL phosphate buffer saline (PBS, pH 7.4) with mild agitation at 75 rpm [45]. To maintain a consistent volume, aliquots were removed at 1, 2, 3, 4, 5 and 6 h and replenished with 2 mL of fresh medium. A UV spectrophotometer set at λ_max_ 283 nm was used to determine the concentration of ZMP in the withdrawn samples. The experiments were performed in triplicate and the mean percentages released ± SD were calculated and plotted against their respective time intervals. The permeation of ZMP solution was carried out simultaneously to validate that the cellulose membrane is not a delaying factor for drug permeation [32]. Models of zero, first and Higuchi diffusion [46] were used to fit the drug release profiles. The best-fitting model was the one with the highest coefficient of determination (R^2^). 

##### Desirability and Optimization

In order to statistically optimize the composition of the prepared NLCs, the desirability values were computed to determine the formula with the lowest PS and PDI and the highest ZP, EE and Q6h. The desirability value integrates all of the dependent replies into a single assessment that determines the superlative values of the independent parameters. It is calculated separately for each desirability function [32].

##### Thermal Properties Utilizing Differential Scanning Calorimetry

Differential scanning calorimetry (DSC) is an important tool to study the thermal properties of different excipients used and the drug. It can also give an insight into and confirm the entrapment of ZMP. DSC thermograms of Compritol^®^, Miglyol^®^, Span 20, ZMP and the candidate lyophilized NLC9 ((Vacutec freeze drier, Labcinco^®^, Corp., Kansas City, MS, USA, no cryoprotectant) were recorded using a thermal analyzer (TA-60, Shimadzu, Japan). The samples were hermetically sealed in aluminum pans and heated at a constant rate of 10 °C/min over a temperature range of 25 to 300 °C.

##### Morphological Visualization of the Candidate Formula (NLC9)

The candidate nanostructure lipid carrier (NLC9) was examined for morphological appearance and aggregation with the aid of transmission electron microscope (TEM; Jeol, 100 CX-TEM, Tokyo, Japan). The prepared formula was diluted with double distilled water and dropped on a copper grid. Then, a drop of 0.2% phosphotungistic acid was added to stain the formula. The copper grid was then left to be air-dried for 5 min and examined under TEM utilizing an accelerating voltage of 80 K.

#### 2.2.4. Preparation of ZMP-NLC Loaded Gels

The optimized formula (NLC9) was utilized to prepare gels intended for transdermal delivery of ZMP. Incorporation of nanoformulations into topical gels increased their stability, with easier spreadability and application. First, NLC9 was prepared following hot melt emulsification method as mentioned in the “Preparation of ZMP loaded NLCs” section. Two different gelling agents were used to prepare the gel required, namely, hydroxypropyl methyl cellulose (HPMC E15) in 2% *w*/*w* concentration and pemulen^®^ in 0.5% *w*/*w* concentration. Each was added separately utilizing the given concentration to the hot double distilled water. The systems were stirred overnight to ensure the complete dispersion of the gelling agents, resulting in gel consistency [47]. The pH of pemulen^®^ gel was adjusted within the range of 6.8 to 7.2 by adding few drops of triethanolamine [48]. For further characterization, the gels were kept at room temperature.

#### 2.2.5. Characterization of NLC9 Loaded Gel

##### Visual Inspection

Visual inspection of the appearance and other physical aspects of freshly formulated gels, including color, precipitation and homogeneity, was performed against black and white backgrounds.

##### Determination of Drug Content

In a measuring flask, one gram of the produced gels was diluted to 100 mL with methanol. The drug content was then determined spectrophotometrically at λ_max_ 283 after performing the necessary filtration step [49]. The drug content was calculated utilizing the following equation:

$$Drug\;content=\frac{actual\;drug\;amount\;in\;1\;g\;gel}{theoretical\;drug\;amount\;in\;1\;g\;gel\;}\times 100$$

##### Determination of Gel pH

A digital pH meter (Jenway TM 3510, Staffordshire, London, UK) was used to determine the pH of various gel formulations. One gram of the prepared gels was dissolved in 9 mL of distilled water using a magnetic stirrer, warming over water bath if needed. Subsequently, after cooling to room temperature and storage for 2 h, the pH was determined [49].

##### Rheology Study of All Prepared Gels

The viscosity of the two gels prepared (2% HPMC and 0.5% pemulen^®^) loaded with the candidate formula (NLC9) was evaluated using cone and plate viscometer. The freshly formulated gels were positioned in the cup of the viscometer using spindle 52 at 25 °C ± 1. The samples were continuously sheared at various rates.

Measurements were taken across the entire speed range of 25 to 250 rpm [50] with 30 s interval between each two consecutive speeds, and then in the same descending order. The shear rate in s^−1^, the shear stress in dyne/cm^2^ and viscosity in centipoises were calculated. In addition, the viscosities at maximum shear rate (η_max_), at minimum shear rate (η_min_), flow indices (*n* values) and hysteresis loop areas were obtained. The loop areas were calculated by subtracting the area under the down curve from the area under the up curve and dividing it by the used scale to obtain the result in cm^2^ [51].

#### 2.2.6. Ex Vivo Evaluation of NLC9 Loaded Gels

##### Skin Preparation

Ex vivo skin penetration and permeation tests were conducted using albino rat shaved skin purchased from local animal house. A cervical dislocation procedure was used to sacrifice albino rats. Skin tissues were removed from the rat’s dorsal side. The dorsal hair was clipped out with a hair clipper. The shaved region was then separated using a surgical blade No. 23. A scalpel was used to remove subcutaneous fat from the skin immediately after incision. The skin was washed in normal sterile saline (0.9% NaCl solution) and then sectioned into appropriate size pieces for permeation testing [52].

##### Skin Permeation Procedures

The USP dissolution apparatus II was used to investigate the ex vivo skin penetration and permeation of several NLC9-loaded gels. Fresh dorsal rat skin pieces with a permeation area of 3.14 cm^2^ and a receptor cell volume of 50 mL were fixed between the donor and receptor compartments. The fixed skin was stranded on a receptor compartment with the donor compartment facing the stratum corneum side. The receptor compartment was filled with 50 mL of phosphate buffer saline (PBS, pH 7.4) and held at 35 ± 0.5 °C under constant stirring at 75 rpm for 6 h. Aliquots of 2 mL were taken from the receptor compartment at 1, 2, 3, 4, 5 and 6 h and replaced with fresh medium. Determination of the amount of ZMP permeated was carried out adopting HPLC technique according to the method developed by Danavena et al. [53]. Their method was adapted and validated to our lab with a lower limit of quantitation at 60 µg/mL. A linear calibration curve was obtained between 60–100 µg/mL [54,55]. The ex vivo permeation of gels prepared containing the same amount of drug was carried out simultaneously for comparison reasons. Average cumulative amount of ZMP permeated through the skin surface per unit surface area (μg/cm^2^) was quantified and displayed as a function of time. The permeability (Pc), steady state flux (Jss; μg/cm^2^·h) and diffusion (D) coefficients were then computed as permeation parameters.

##### Skin Deposition

After the permeation study was accomplished, the skin was removed and the surface was cleaned three times with PBS (pH = 7.4). The skin samples were separately soaked for 24 h in a flask containing 20 mL of methanol. The methanolic samples were then sonicated in ultrasonic bath for four cycles (30 min each) to extract all ZMP stored in the skin parts. After that, the methanolic solution was filtered and analyzed using the same HPLC procedure.

#### 2.2.7. Histopathological Examination

To assess the possible irritating impact of the developed formula, the optimum zolmitriptan NLCs loaded gel was smeared to the shaved dorsal skin of a rabbit in an area of 5 cm^2^ diameter for three consecutive days. The rabbit was then slaughtered, and skin sample was taken and preserved in formalin solution (10%). The sample was then desiccated with ethanol, fixed in paraffin and stained with hematoxylin and eosin. Five µm microtome sections were cut and inspected under a light microscope (National model 138, Guilin FT-OPTO Co., Ltd., Guilin, China) [32].

#### 2.2.8. In Vivo Pharmacokinetic Study

##### Animals

The study included six male New Zealand rabbits (2–3 kg). Each rabbit was kept in its own cage, with humidity and temperature adjusted at 55 ± 5% and 22 ± 2 °C, respectively, in order to keep them healthy. Equal daily cycles of light and dark conditions were maintained, according to the *Guide for the Care and Use of Laboratory Animals*.

##### Study Design

With an application number of PI 2653, the study design was evaluated and authorized by the ethics committee of Faculty of Pharmacy, Cairo University, Cairo, Egypt.

The rabbits were allocated arbitrarily into two groups. The first group received zolmitriptan marketed product (Zomig^®^) orally and the second group received the NLC9-HPMC gel applied to the rabbit’s shaved dorsal skin at a dose of 0.9 mg/kg [56]. To remove subject-to-subject variance, a randomized two-period crossover design with a washout interval of a week was adopted [32]. 

Blood samples of 2 mL were taken from the tail vein after oral treatment or topical application at specified time intervals (0.5, 1, 2, 4, 6, 8 and 24 h). Plasma was separated by centrifugation at 4000 rpm for 20 min at 5 °C, then kept at −20 °C until analysis.

##### ZMP Determination in Plasma

An internal standard, torsemide (100 ng/mL), was combined with a 100 μL alcoholic solution of plasma samples that had been thawed at room temperature. Extraction of ZMP from plasma samples was carried out by vortexing 0.5 mL plasma with 4 mL of tertiarybutyle methyl ether. The organic solvent was then evaporated under vacuum and the samples were reconstituted with 0.5 mL of the mobile phase, which was composed of 4:1 *v*/*v* acetonitrile: 0.1% formic acid. The samples were injected into a Shimadzu LC/MS/MS (Tokyo, Japan) with a Sunfire column (C18, 4.6 × 50 mm, Waters Corporation, Milford, MA, USA) and a mobile phase flowing at 0.8 mL/min. The apparatus functioned with a cationic approach, a dwell of 500 ms and scan speed of 10 Da/s. The declustering potential and precursor-to-product ion ratios were 51, 287.6/58 and 96,349.1/263 for ZMP and torsemide, respectively. Linearity (R^2^ = 0.9997) and accuracy (R^2^ = 0.9998) were used to validate the analysis approach, with a lower limit of detection of 0.01 ng/mL.

##### Pharmacokinetic Analysis

Kinetica^®^ software (Version 5, Thermo Fisher Scientific Inc., Minneapolis, MN, USA) was employed to analyze the obtained in vivo results utilizing non compartmental analysis technique to determine key pharmacokinetic parameters: the ZMP maximum concentration in plasma (C_max_), its corresponding time (T_max_), the area under the plasma concentration versus time curve from time zero to the 24 h (AUC_0→24_), the area under the plasma concentration versus time curve from time zero to infinity (AUC_0→∞_) and the relative bioavailability compared to the ZMP marketed product (Zomig^®^). The results were expressed as mean value of six rabbits ± S.D. Student′s *t*-test created by SPSS 19^®^ software was used to statistically examine the estimated pharmacokinetic parameters. Statistical significance was defined as a *p*-value of less than 0.05.

## 3. Results

Since the first NLCs were fabricated, they started to be known as the second generation of lipid nanocarriers. They comprise one or more solid lipid and liquid lipid blends. It was also shown that NLCs are maintained in a solid state in the body [57]. NLCs contain liquid lipid blends as core-forming materials that lead to a decrease in their melting point. However, this occurs without affecting the physical state of the formed lipid carriers [58]. A 3^2^ full factorial design was employed to study the effect of two variables, each in three levels. The study design was generated and analyzed by Design Expert^®^ software. The nine NLCs formulae were successfully prepared adopting hot melt emulsification followed by high-speed stirring and ultrasonication. Statistical analysis was carried out either using main effect or two-factor interaction at a *p*-value < 0.05. All interpretations showed an adequate precision, higher than 4, with a small difference between the adjusted R^2^ and predicted R^2^ (<0.2) (Table 3) [59]. The lack of fit in all scrutiny was statistically insignificant, indicating the properness of the model to navigate the responses [32].

The results of the measured responses are given under the following headings:

### 3.1. Characterization of ZMP Loaded NLCs

#### 3.1.1. Analysis of PS Results

It has always been postulated that nanoparticles, possessing the ability to transfer the drug beyond the epidermis and dermis, allow for the pharmacological moiety to reach the systemic circulation and to exert its pharmacological effect on what is commonly known as transdermal absorption [60]. NLCs have been successfully used to achieve transdermal drug delivery, owing to their small particle size (nano range) [61], in addition to their pronounced lipophilic nature that enhances the interaction with the lipid bilayers of the epidermis and dermis [62].

**Figure 1:**
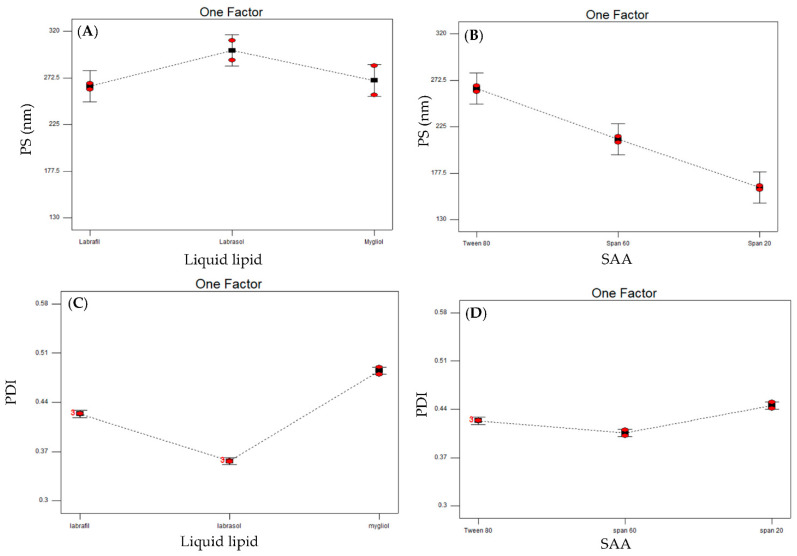
Line plots of the effect of the two significant studied variables: liquid lipid type on particle size (**A**), surfactant type on particle size (**B**), liquid lipid on polydispersity index (**C**) and surfactant type on polydispersity index (**D**).

The measured PS was in the nano scale, ranging from 138.3 nm (NLC9) to 300.4 nm (NLC2) (Table 2). The results were subjected to statistical analysis utilizing a two-factor interaction model with an adequate precision of 16.23 (˃4) and small difference (<0.2) between the adjusted R^2^ (0.9314) and predicted R^2^ (0.854) (Table 3). The model was found to be statistically significant for both of the independent variables investigated (*p* < 0.001). The model graphs of the two-factor interaction statistical analysis revealed that Labrasol^®^ possessed a higher PS in comparison to the other used lipids (Labrafil^®^ and Mygliol^®^) (Figure 1A). These results are in line with the findings declared by Sylvie et al. [63] who found that the colloidal properties of 1% Labrasol^®^ enhances the formation of higher PS particles that reached up to 1000 nm and that, the higher the concentration of the lipid, the higher the measured PS. In addition, regarding the insights towards molecular weights (Mw) of liquid lipids used, Labrasol^®^ is considered to be the highest Mw amongst them. Thus, its incorporation in the formed NLCs would favor the formation of particles with a higher size in comparison to Labrafil^®^ and Mygliol^®^. On the other hand, it is worth mentioning that Labrasol^®^ has the highest viscosity (80–110 cp) in comparison to Labrafil^®^ (70–90 cp) and Mygliol^®^ (30 cp). Increasing the viscosity would eventually hinder the ability of ultrasonication to decrease the particle size of the formulated NLCs, resulting in bigger particles.

In terms of SAA used as stabilizers in the formed NLCs, Tween 80 showed a significantly higher PS ˃ Span 60 ˃ Span 20 (Figure 1B). These results are in line with the investigations by Fatemeh et al. [64], who declared that niosomes prepared comprising Tween 80 showed a higher PS than those prepared using Span 60. Their results proved that the higher the HLB value of a SAA, the higher the particle size. Increasing the HLB value of the SAA would increase the molecule free energy due to the incorporation of more hydrophilic groups, therefore yielding a higher PS [65]. Among the SAA used, Tween 80 has the highest HLB value (15), whereas those of Spans used are between 5 and 9. 

It is also worth noting that the interaction of the two factors studied, namely, liquid lipid type and SAA type, was found to be statistically significant (*p* < 0.0001), where NLCs prepared using Tween 80 showed a higher PS in all of the liquid lipids used. Tween 80 has both the highest HLB value and the highest Mw among the SAA employed in NLCs preparations.

#### 3.1.2. Analysis of PDI Results

The polydispersity index (PDI) is a parameter used to describe the size range of the lipidic nanocarriers [66]. The measured values could be any denominations between 0 and 1, where lower values indicate more homogenous nanodispersions than higher values. Usually, values < 0.5 and considered acceptable concerning the homogeneity of the prepared dispersions [59]. The measured PDI values ranged from 0.3095 (NLC9) to 0.578 (NLC 5) (Table 2).

Statistical optimization was carried out employing two-factor interaction with an adequate precision of 83.979, adjusted R^2^ and predicted R^2^ values of 0.9963 and 0.9922, respectively (Table 3). Both of the studied variables, either liquid lipid type or SAA type, were found to be statistically significant (*p* < 0.0001). Model graphs revealed that Labrasol^®^ had a lower PDI than Labrafil^®^ and Mygliol^®^ (Figure 1C). The results can be explained in light of the viscosity of the different liquid lipids used. Increasing the viscosity would hinder the ability of the probe sonication to decrease the PS of the nanoparticles prepared, as discussed earlier under PS interpretation. Failure to decrease the PS by sonication would keep the NLCs in the original PS, offering more homogenous systems and eventually decreasing the measured PDI, shifting the results to lower values. Similar results were aroused by Safwat et al. [67], who declared that increasing the medium viscosity will ultimately increase the system homogeneity. 

On the other hand, Figure 1D disclosed that Span 20 had significantly higher PDI values in comparison to the other two SAA. Similar results were declared by Abdel Fadeel et al. [68], whose work asserted that lipid carriers prepared utilizing Spans showed a higher PDI in comparison to Tweens. The incorporation of low-molecular-weight SAA (Span 20, Mw 346.6) with shorter alkyl chains in the membrane of the formed nanoparticles would cause less dense packing [69] and decrease the envelop rigidity in comparison to higher-molecular-weight SAA (Span 60, Mw 430.62 and Tween 80, Mw 1310) [70]. Decreasing the membrane rigidity would lead to a profound effect of probe sonication, used during the preparations of NLCs, resulting in a greater fragmentation of the prepared NLCs and higher PDI values. 

#### 3.1.3. Analysis of ZP Results

**Figure 2:**
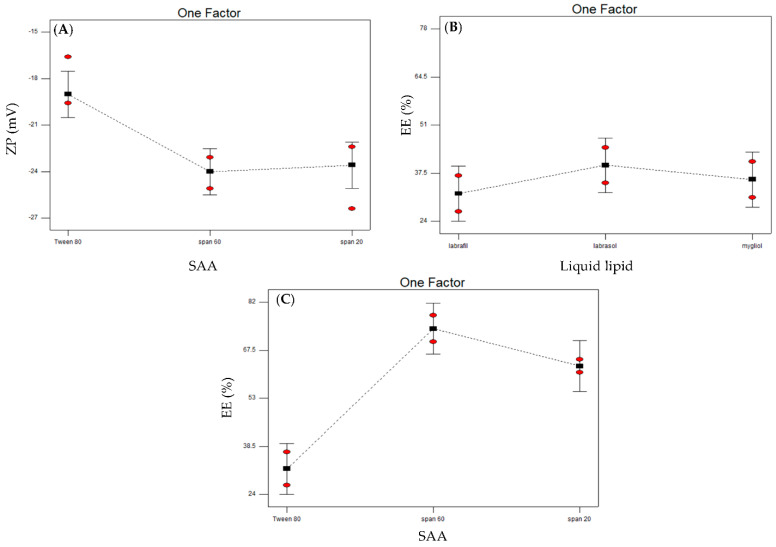
Line plots of the effect of the two significant studied variables: surfactant type on zeta potential (**A**), liquid lipid type on entrapment efficiency (**B**) and surfactant type on entrapment efficiency (**C**).

Charges acquired by the prepared NLCs were assessed utilizing the ZP term. The measured ZP values ranged from −17.9 (NLC3) to −24.4 (NLC7) (Table 2), indicating that some formulae have better stability (higher than 20) than other formulae (lower than 20) [43]. It is worth noting that all prepared NLCs developed negative ZP. This can be assigned to the presence of acidic groups in both the solid lipid (Compritol^®^) [71] and liquid lipids (oils) [72,73] used. The results were subjected to statistical analysis employing the main effects model with an adequate precision of 6.54 and a small gap between the adjusted R^2^ (0.5904) and the predicted R^2^ (0.3995) (Table 3). The model was found to be statistically significant (*p* < 0.005), with an insignificant lack of fit (*p* = 0.8729). Differentiating the main effects of the studied variables revealed that the liquid lipid type affected the measured ZP insignificantly (*p* ˃ 0.01), whereas the SAA type showed a significant effect on ZP (*p* < 0.001). Tween 80 showed higher ZP values in comparison to Spans (20 and 60) used (Figure 2A). These results can be explained regarding HLB values, where Hong et al. [74] believed that microemulsions prepared with HLB values above 11 have higher ZP in comparison to those prepared with lower HLB values (less than 10). This may be due to the adsorption of OH^-^ species from the aqueous medium [75], in addition to the higher ability of Tween 80 to form hydrogen bonds between the polyoxyethylene group and OH^−^, resulting in the formation of oxonium ions [76].

#### 3.1.4. Analysis of EE Results

The measured EE of ZMP-loaded NLCs ranged from 31.7% (NLC1) to 73.9% (NLC4) as shown in Table 2. The statistical analysis was conducted employing a two-factor interaction model that was found to be statistically significant (*p* < 0.0008). The model had an acceptable adequate precision value of 8.789, with a small gap between the adjusted R^2^ (0.8253) and predicted R^2^ (0.6301) (Table 3). The liquid lipid type was found to be statistically significant, with a *p* value of 0.0326. Interpreting the model graphs revealed that Labrasol^®^ had a higher EE in comparison to the other liquid lipids used, namely, Labrafil^®^ and Mygliol^®^ (Figure 2B). These results coincide with the PS measurements, where increasing the PS would eventually lead to entrapping more of the drug. Similar results were reached by Mohanty et al. [77], who formulated naproxen as transdermal niosomes. The EE results interpretation revealed that the prepared nanoparticles entrap more of the drug as their size increases. 

On the other hand, Span 60 as a SAA used in the formulation of NLCs showed a higher EE in comparison to the other two SAA used, whereas Tween 80 had the lowest EE (Figure 2C). The statistical analysis of the two-factor interaction model was found to be significant, with a *p*-value of 0.0003. Studying the Tween 80 structure revealed the presence of a high degree of unsaturation, allowing the alkyl chains to bend. This bending can decrease the NLCs closure and stiffness, increasing the permeability of the formulated nanoparticles and leading to a lower drug entrapment [78]. On the other hand, the reason why Span 20 led to a lower EE in comparison to Span 60 could possibly be due to the shorter alkyl chain, which leads to the formation of a less dense hydrophobic monolayer accommodating a lower amount of the hydrophobic ZMP [70].

#### 3.1.5. Analysis of Q6h Results

**Figure 3:**
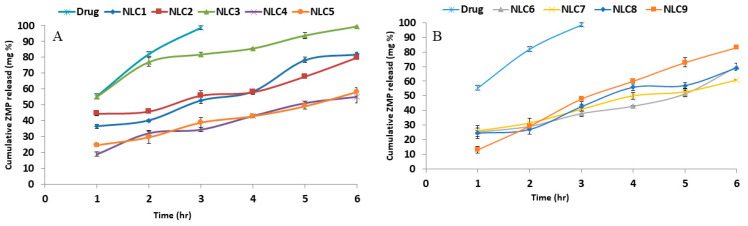
In vitro ZMP release profiles from the prepared NLCs (**A**) and drug solution in PBS (pH 7.4) at 35 ± 0.5 °C (**B**), mean ± SD, *n* = 3.

**Figure 4:**
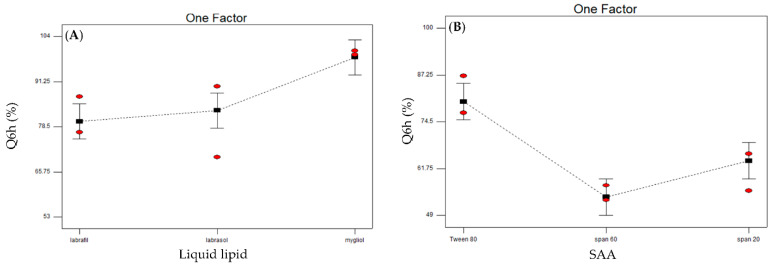
Line plots of the effect of the two significant studied variables: liquid lipid type (**A**) and surfactant type (**B**) on in vitro release.

The ZMP release from the developed NLCs is depicted in Figure 3. The permeation of the drug solution was carried out first through the dialysis surface to make sure that the dialysis membrane has no retardation effect on drug diffusion. The drug solution reached 100% in 3 h, signifying the absence of any interaction between the drug and the dialysis membrane. On the other hand, drug release profiles from the prepared NLCs are a crucial step in their characterization, as they are used to anticipate and predict drug behavior in vivo. The amount of the drug released after 6 h (Q6h) is compiled in Table 2. It is worth noting that the prepared formulae had a slower release rate than drug permeation, where some NLCs can maintain a drug release of more than 6h. A statistical analysis of the results was investigated utilizing the main effects model. Both studied factors were found to be statistically significant, with an insignificant lack of fit. The calculated predicted R^2^ was 0.7430, with a small gap to the adjusted R^2^ (0.8237) and an adequate precision of 13.37 (Table 3). Model graphs showed that Mygliol^®^ had a statistically higher Q6h value in comparison to the other two liquid lipids used (Figure 4A). This can be explained in terms of PS, as Mygliol^®^ showed a lower PS in comparison to Labrasol^®^. A lower PS would increase the exposed total surface area of the prepared NLCs, enhancing the drug dissolution and release from the prepared nanoparticles. Similar results were revealed by Abdel-Rahim M. El-Helw and Usama A Fahmy [79], who formulated NLCs containing the drug fluvastatin. An interpretation of release data showed that, the lower the particle size measured, the higher the surface area of the nanoparticles prepared, and, consequently, the higher the release rate. On the other hand, among the SAA used, Tween 80 showed a higher Q6h in comparison to Span 60 and Span 20 (Figure 4B). This can be justified in terms of EE. Tween 80 showed the lowest EE of the prepared NLCs. The lower the drug entrapment in the prepared particles, the faster the release rate [80,81]. The in vitro release profiles of some formulae were best fitted to the Higuchi diffusion model (NLC3 and NLC5), whereas the others were fitted to the zero order model.

### 3.2. Selection of the Candidate NLC Formula

Design Expert^®^ software was utilized to calculate the desirability functions for the prepared formulae. The required constraints were to minimize both the PS and PDI and to maximize ZP, EE and Q6h (Table 1). It is known that decreasing the nanoparticles size and PDI values will improve the skin penetration ability [32] and homogeneity [59] of the formulated systems, respectively. On the other hand, maximizing ZP will enhance the nanoparticles stability [59] by increasing the interparticle repulsion, which eventually retards system sedimentation. Increasing EE will favor the drug entrapment in the formulated NLCs and decrease the unentrapped drug; hence, increasing the drug skin permeation abilities. In addition, increasing Q6h will accelerate the drug to reach therapeutic levels and exert its pharmacological effect. NLC9 was suggested by the software with a desirability value of 0.737. The formula contained Mygliol^®^ (1%) as the liquid lipid component and Span 20 (1%) as a SAA. The measured variables were 138.3 nm for PS, 0.3095 for PDI, −22.17 for ZP, 73% for EE and 82.01% for Q6h (Table 2).

### 3.3. DSC Results

**Figure 5:**
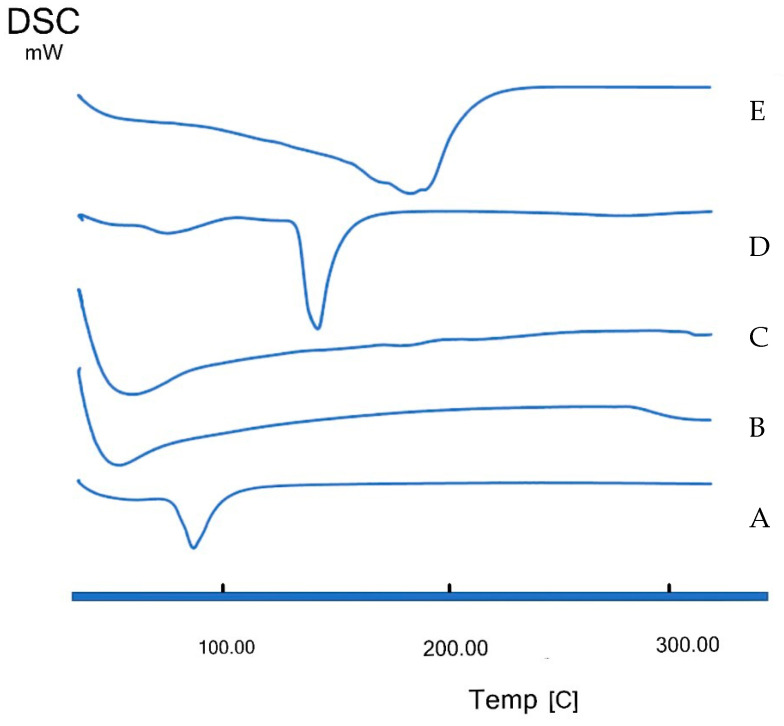
DSC thermograms of Compritol^®^ (**A**), Miglyol^®^ (**B**), Span 20 (**C**), ZMP (**D**) and NLC9 (**E**) at a heating rate of 10 °C/min up to 300 °C.

DSC thermograms are presented in Figure 5. All of the studied excipients, namely, Compritol^®^, Miglyol^®^ and Span 20, showed their corresponding phase transition temperatures (Figure 5A–C). ZMP showed a melting point at 141 °C, indicating drug purity [16] (Figure 5D). The drug endothermic peak disappeared in NLC9 (Figure 5E), which suggests the incorporation of the drug in the prepared NLCs nanocarriers turning it into an amorphous state with no sharp endothermic peak but, rather, a melting range. In addition, a very broad peak was observed that was initiated at 40.24 °C and ended at 244.4 °C, with a peak maximum at 183.67 °C, indicating the interaction between the solid lipid (Compritol^®^), liquid lipid (Miglyol^®^) and SAA used (Span 20) [82].

### 3.4. Morphological Examination of NLC9

**Figure 6:**
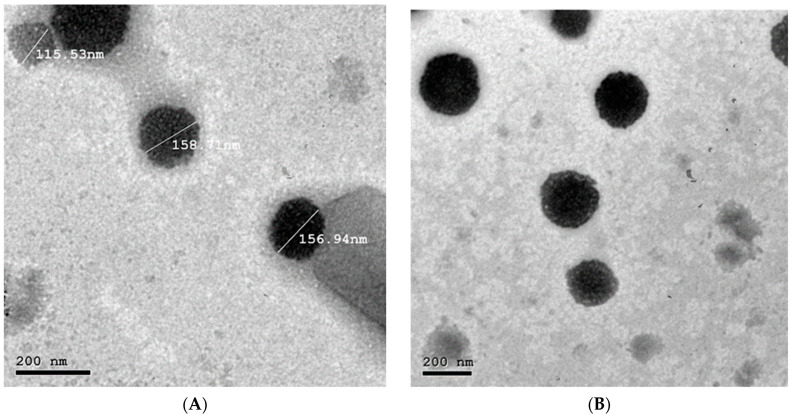
Transmission electron microscope image of the optimized zolmitriptan NLC (NLC9), (**A**), with scale, (**B**), without scale.

The TEM micrographs showed that the prepared NLCs are spherical in shape and well-defined with no aggregation (Figure 6A,B). The particles revealed a consistent size with that measured utilizing the light scattering dynamic approach (Figure 6A). The integrity of the particles was not compromised by the sonication stage, since no structural abnormalities were identified (Figure 6B). Similar results were declared by Marianne and Amal [83], who prepared flibanserin nanoparticles utilizing probe sonication for 10 min; however, the prepared formulae maintained its structure without disruptions. 

### 3.5. Characterization of NLC9 Loaded Gel

Due to the lower viscosity of aqueous dispersions, NLCs are not sufficiently physically stable in liquid form, making them challenging to be utilized topically. Furthermore, NLCs aqueous dispersions are not stable for a long period of time, and certain systems display particle aggregation or an increase in particle size. However, by incorporating the prepared NLCs dispersion into a gel basis, the nanoparticulate structure can be preserved, and aggregation of the particles can be prevented. The prepared gels were translucent, with a smooth consistency and no grittiness in touch. Visually, there were no particles or clumps observed. The measured pHs of the gels were between 6.5–7, which is near to the physiological pH of the skin, eliminating the likelihood of irritation or inflammation. The drug content was 96.6% and 91.1% for HPMC and pemulen^®^ gels, respectively.

### 3.6. Rheology Study of the Prepared Gels

**Figure 7:**
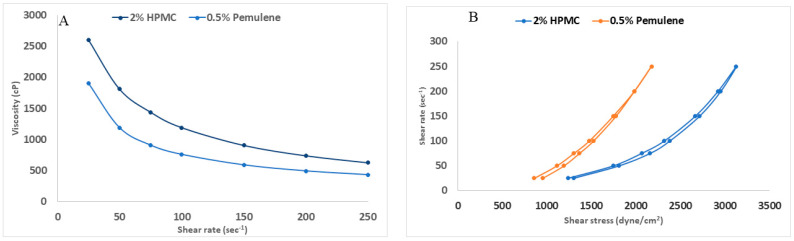
Rheological parameters of the prepared zolmitriptan NLCs loaded gels: viscosity determination (**A**) and hysteresis loop measurement (**B**).

The results of viscosity measurements of the prepared gels are displayed in Figure 7. Both nanovesicular gels prepared had a maximum viscosity (η_max_) of 2604 and 1905 cp for HPMC and pemulen^®^ gels, respectively (Figure 7A). The viscosity abruptly decreased to reach a value of 625.5 and 435 cp (η_min_) for HPMC and pemulen^®^ gels, respectively (Figure 7A). This indicated a shear thinning behavior, where the viscosity of the systems decreases with an increasing shear rate. Both HPMC and pemulen^®^ have been reported to result in the formation of shear thinning gels [84,85]. The use of shear thinning systems is always favorable for topical application. Decreasing the viscosity upon shears stress application—rubbing—will ease the spreadability of the gels on the skin [32]. 

Plotting the shear rate versus shear stress (Figure 7B) confirmed that the prepared gels had a pseudoplastic flow, with flow indices (n value) of 0.6231 and 0.6399 for HPMC and pemulen^®^ gels, respectively. It is well known that the flow index is lower than 1 in the case of pseudoplastic systems [32].

The rheological properties of thixotropic systems can be studied via hysteresis experiments. The approach entails determining the areas encompassed between the up curve rheogram, which corresponds to increasing shear rates, and the down curve rheogram, which corresponds to lowering shear rates after agitation durations. The area of the hysteresis loop is used as a quantitative measure of thixotropy, where, the greater the hysteresis loop area, the greater the increase in the thixotropy [86]. The hysteresis loop areas were 1.1937 and 0.9355 cm^2^ for HPMC and pemulen^®^ gels, respectively, indicating the higher thixotropic powers of the HPMC gel. This was an initial reason to give the HPMC gel an added privilege over pemulen^®^ gel.

### 3.7. Ex Vivo Evaluation of NLC9 Loaded Gels

**Figure 8:**
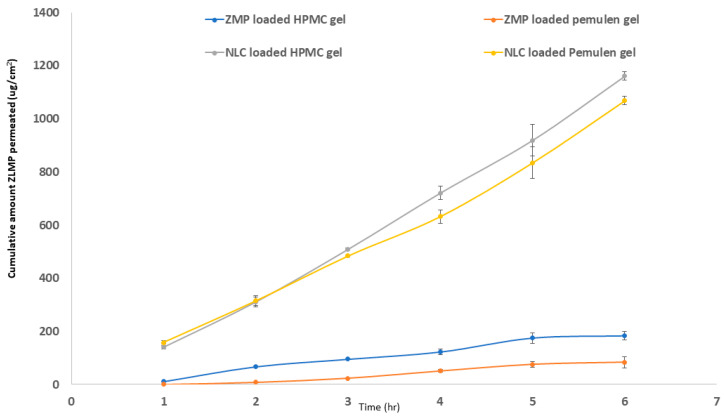
Mean cumulative zolmitriptan permeated from optimized NLCs (NLC9) compared to raw drug suspension, both loaded in HPMC and pemulen^®^ gels, across freshly excised shaved dorsal rat skin.

The ex vivo permeation test is believed to be a valuable tool to provide an acumen to the performance of the prepared gels in vivo. Cumulative ZMP infiltrated per unit area from NLC9-loaded gels compared to the corresponding drug loaded gels are depicted in Figure 8 and the computed permeation parameters are displayed in Table 4. The ex vivo results showed the superior skin penetration powers of the NLCs prepared—either in HPMC or pemulen^®^ gels—in comparison to the corresponding drug-loaded gels. The NLCs-loaded gels revealed a significantly higher maximum amount of drug permeated (D_max_) and steady-state flux (J_ss_) compared to the same gel base loaded only with ZMP, with a 9.6-fold and 5.9-fold increase in the permeability coefficient (Pc) for pemulen^®^ and HPMC gels, respectively. This clarifies the ability of the formulated lipid carriers to overcome the skin barrier properties and allow for the transdermal drug absorption, leading to the systemic delivery of the drug. These results are in line with the results declared by Imran et al. [87], who formulated nanostructured lipid carriers containing both quercetin and resveratrol. Their results displayed the higher skin penetration powers of the formulated NLCs.

On the other hand, the statistical analysis of HPMC gels—both loaded with NLC9 and pure drug—disclosed that the HPMC group had higher transdermal drug delivery abilities in comparison to pemulen^®^. This is further justified by the higher D_max_, J_ss_ and permeability coefficient (Pc) calculated for the HPMC group. Regarding the structure of HPMC E13 used in the preparation of the HPMC gel, it is revealed that the molecule has a degree of saturation (DS) ≈ 2. This property increases the hydrophobic characteristics of the formed gel, which eventually facilitates the transdermal delivery of the loaded drug [88]. This finding, together with the greater thixotropic properties of the HPMC gel, favors its selection for the subsequent in vivo assessment.

### 3.8. Histopathological Examination

**Figure 9:**
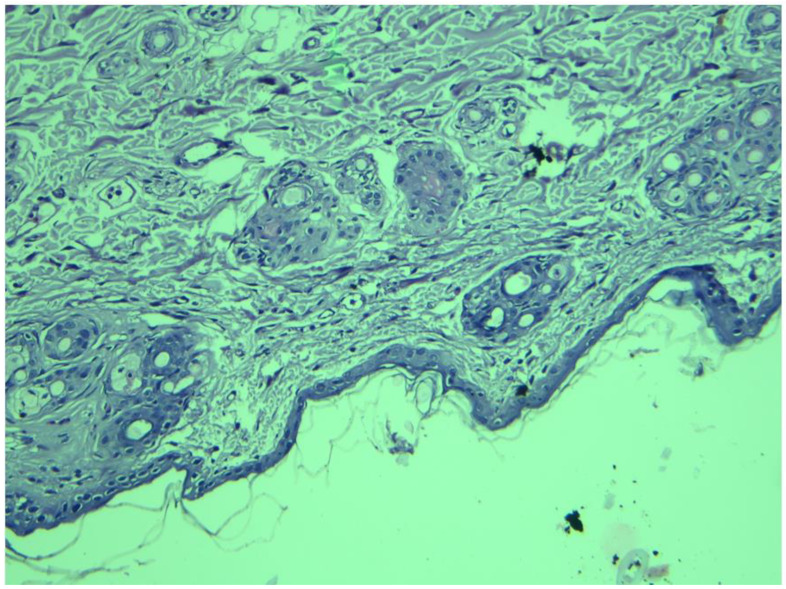
Histopathological examination of shaved rabbit dorsal skin treated with NLC9-loaded HPMC gel for three consecutive days (100×).

To predict any possible irritating effect of the applied zolmitriptan NLC9-loaded HPMC gel, a histopathological examination was performed on a shaved dorsal rabbit skin. Normal squamous epithelium, epidermis and underlying dermis with typical adnexa were noticed (Figure 9). There were no symptoms of inflammatory cell infiltration, skin irritation or necrosis. These findings ratified the safety of the developed zolmitriptan NLC9-loaded HPMC gel for topical application. 

### 3.9. In Vivo Pharmacokinetic Study

**Figure 10:**
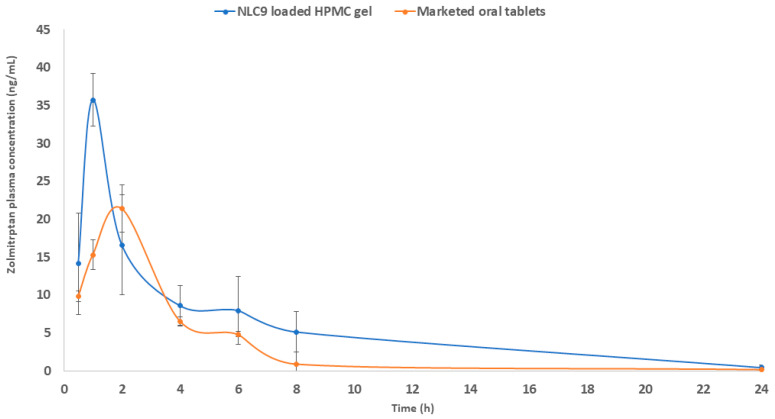
Mean plasma concentrations (ng/mL) of zolmitriptan after transdermal delivery of NLC9-loaded HPMC gel compared to the oral marketed product (Zomig^®^).

To forecast the performance of the formulated NLC9-loaded HPMC gel after topical application, the in vivo test was carried out on three male rabbits in a cross-over design. A cross-over design is frequently used to eliminate noise resulting from inter-subject variations. The in vivo profiles of ZMP administered transdermally to rabbit dorsal shaved skin in the form of NLC9-loaded HPMC gel, in comparison to the oral administration of the ZMP marketed product (Zomig^®^), are presented in Figure 10. The results are expressed as the ZMP plasma concentration versus time as a mean of six rabbits ± SD. The calculated pharmacokinetics parameters are compiled in Table 5. 

The topically applied NLCs showed a statistically higher C_max_, lower T_max_ and higher AUC _0→∞_ (35.7 ng/mL, 1 h, 143.73 ng·h/mL for the NLC9 and 21.4 ng/mL, 2 h, 81.4 ng·h/mL for the oral marketed product, respectively). It is also worth mentioning that the transdermal NLCs showed a relative bioavailability of 176.56%. The above-mentioned results can be explained in light of multiple reasons, namely, the ability of the prepared nanocarriers to evade the first pass metabolism of the drug. The first pass effect is responsible for the decrease in the bioavailability of ZMP to 40%. In addition, the candidate formula (NLC9) had a PS of 138.3 nm. This small PS, in addition to the presence of both liquid lipids and solid lipids, can increase the lipophilic properties of the prepared nanoparticles, increasing the NLC affinity to cross biological barriers [89]. 

## 4. Conclusions

A full factorial design was adopted to prepare and optimize zolmitriptan NLCs. Both studied variables, namely, the liquid lipid type and surfactant type, had a significant effect on the particle size, polydispersity index, entrapment efficiency and amount released after 6 h, whereas the zeta potential was only affected by the surfactant type. The optimized formula (NLC9) showed a minimum particle size (138 ± 7.07 nm), minimum PDI (0.39 ± 0.001) acceptable ZP (−22.1 ± 0.80 mV), maximum EE (73 ± 0.10%) and maximum Q6h (83.22 ± 0.10%). NLC9 was then incorporated into HPMC and pemulen^®^ gels. A rheological examination and ex vivo analysis of the prepared gels revealed more desirable rheological parameters and a higher skin penetration in favor of the HPMC gel, so it was chosen for further in vivo characterization. The pharmacokinetic model conducted in rabbits showed that the prepared NLCs can improve the bioavailability of ZMP by up to 1.76-fold in comparison to the oral marketed product, proving the ability of the developed nanocarriers to penetrate different skin layers and deliver the drug to the systemic circulation. However, more research is required to prove the brain availability of ZMP utilizing the developed NLCs.

## Figures and Tables

**Table 1:** The independent and dependent variables for the 3^2^ full factorial design used for the optimization of ZMP-loaded NLCs formulation.

Factors (Independent Variables)	Levels		
**X_1_:** Liquid lipid type	Labrafil^®^	Labrasol^®^	Mygliol^®^
**X_2_:** SAA type	Tween 80	Span 60	Span 20
**Responses (dependent variables)**	**Desirability constraints**		
**Y_1_:** PS (nm)	Minimize		
**Y_2_:** PDI	Minimize		
**Y_3_:** ZP (mV)	Maximize		
**Y_4_:** EE (%)	Maximize		
**Y_5_:** Q6h (%)	Maximize		

**Table 2:** Experimental runs, independent variables and measured responses of ZMP-loaded NLCs following a 3^2^ full factorial design ^a,b,c^.

Formula Code	X_1_: Liquid Lipid Type ^d^	X_2_: Surfactant Type ^e^	Y_1_: Particle Size (nm)	Y_2_: Polydispersity Index	Y_3_: Zeta Potential (mV)	Y_4_: Entrapment Efficiency (% *w*/*w*)	Y_5_: Q6h (%)
NLC1	Labrafil^®^	Tween 80	263.9 ± 4.24	0.423 ± 0.001	−18.1 ± 2.12	31.7 ± 1.07	81.95 ± 1.10
NLC2	Labrasol^®^	Tween 80	300.4 ± 1.41	0.356 ± 0.001	−18.8 ± 2.82	39.7 ± 3.23	79.85 ± 1.14
NLC3	Miglyol^®^	Tween 80	269.9 ± 2.12	0.485 ± 0.007	−17.9 ± 2.28	35.7 ± 0.40	99.32 ± 0.40
NLC4	Labrafil^®^	Span 60	212.0 ± 4.24	0.406 ± 0.005	−24.1 ± 1.41	73.9 ± 3.90	55.14 ± 3.90
NLC5	Labrasol^®^	Span 60	219.0 ± 4.07	0.578 ± 0.002	−23.3 ± 0.50	42.7 ± 4.24	58.21 ± 2.82
NLC6	Miglyol^®^	Span 60	282.4 ± 2.28	0.425 ± 0.007	−22.4 ± 1.18	41.3 ± 2.83	69.89 ± 1.20
NLC7	Labrafil^®^	Span 20	162.8 ± 0.77	0.445 ± 0.006	−24.4 ± 1.18	62.7 ± 2.50	60.72 ± 2.50
NLC8	Labrasol^®^	Span 20	230.0 ± 3.28	0.392 ± 0.001	−22.0 ± 0.71	48.3 ± 0.30	69.12 ± 0.30
NLC9	Miglyol^®^	Span 20	138.3 ± 7.07	0.309 ± 0.001	−22.1 ± 0.80	73.0 ± 0.10	83.22 ± 0.10

Abbreviations: SAA, surfactant; PS, particle size; PDI, polydispersity index; ZP, zeta potential; EE, entrapment efficiency; and Q6h, amount released after 6 h. ^a^ All the formulae prepared utilizing 12.5 mg of zolmitriptan; ^b^ Values are expressed as mean ± SD; *n* = 3; ^c^ All formulae contained Compritol^®^ as a solid lipid part; ^d^ All liquid lipid types are presented as 1% of the formula; ^e^ All surfactant types are presented as 1% of the formula.

**Table 3:** Regression analysis of the observed responses according to the best fitting model.

Response	Model	R^2^	Adjusted R^2^	Predicted R^2^	Precision	Significant Factors
**Y_1_:** PS (nm)	Two-factor interaction	0.9637	0.9314	0.8547	16.23	**X_1_, X_2_, X_1_X_2_**
**Y_2_:** PDI	Two-factor interaction	0.9947	0.9963	0.9922	83.97	**X_1_, X_2_, X_1_X_2_**
**Y_3_:** ZP (mV)	Main effects	0.6868	0.5904	0.3995	6.54	**X_2_**
**Y_4_:** EE (%)	Two-factor interaction	0.9075	0.8253	0.6301	8.78	**X_1_, X_2_, X_1_X_2_**
**Y_5_:** Q6h (%)	Main effects	0.8660	0.8237	0.7430	13.37	**X_1_, X_2_**

Abbreviations: SAA, surfactant; PS, particle size; PDI, polydispersity index; ZP, zeta potential; EE, entrapment efficiency; and Q6h; amount released after 6 h.

**Table 4:** Ex vivo permeation parameters of optimized zolmitriptan-loaded NLC gels compared to zolmitriptan suspension.

Formulation	D_max_ (µg) ± SD	Jss (µg/cm^2^h)	Pc (cm/h)	D (cm^2^/h)	Deposition (µg/cm^2^)
ZMP-loaded HPMC gel	575.83 ± 47.10	34.54	0.0142	0.0019	74.39
ZMP-loaded pemulen^®^ gel	262.38 ± 62.80	18.47	0.0076	0.0005	64.38
NLC9-loaded HPMC gel	3645.13 ± 109.90	203	0.0838	0.0656	55.25
NLC9-loaded pemulen^®^ gel	3355.5 ± 49.17	178	0.0735	0.0502	50.48

Abbreviations: D_max_, maximum amount of drug permeated; J_ss_, steady-state flux; Pc, permeability coefficient; D, diffusion coefficient; SD, standard deviation.

**Table 5:** Pharmacokinetic parameters of zolmitriptan after transdermal administration of NLC9-loaded HPMC gel in comparison to marketed oral tablets (Zomig^®^) ^a,b^.

Pharmacokinetic Parameters	Marketed Oral Tablets	NLC9-Loaded HPMC Gel
C_max_ (ng/mL)	21.4 ± 3.11	35.7 ± 4.03
T_max_ (h)	2	1
AUC_0–24_ (ng·h/mL)	80.46 ± 23.04	141.18 ± 44.41
AUC_0–∞_ (ng·h/mL)	81.40 ± 23.01	143.73 ±19.70
K (h^−1^)	0.15 ± 0.02	0.161 ± 0.09
T_½_ (h^−1^)	4.40 ± 0.51	4.29 ± 0.48
Relative bioavailability (%)		176.56%

^a^ The dose administered was 0.9 mg/Kg.; ^b^ Values are expressed as mean ± SD; *n* = 6.

## Data Availability

Not applicable.
